# ND-13, a DJ-1-Derived Peptide, Attenuates the Renal Expression of Fibrotic and Inflammatory Markers Associated with Unilateral Ureter Obstruction

**DOI:** 10.3390/ijms21197048

**Published:** 2020-09-24

**Authors:** Carmen De Miguel, Abigayle C. Kraus, Mitchell A. Saludes, Prasad Konkalmatt, Almudena Ruiz Domínguez, Laureano D. Asico, Patricia S. Latham, Daniel Offen, Pedro A. Jose, Santiago Cuevas

**Affiliations:** 1 Section of Cardio-Renal Physiology and Medicine, Division of Nephrology, Department of Medicine, University of Alabama at Birmingham, AL 35233, USA; abkraus@uab.edu; 2 Department of Medicine, Division of Renal Diseases & Hypertension and Pharmacology/Physiology, The George Washington University School of Medicine and Health Sciences, Washington, DC 20052, USA; mtsaludes@live.com (M.A.S.); prk@email.gwu.edu (P.K.); lasico@email.gwu.edu (L.D.A.); pjose@mfa.gwu.edu (P.A.J.); 3 Molecular Inflammation Group, Biomedical Research Institute of Murcia (IMIB), University Clinical Hospital Virgen Arrixaca, 30120 Murcia, Spain; almuruiz_8@hotmail.com; 4 Pathology and Internal Medicine The George Washington University School of Medicine and Health Sciences, Washington, DC 20052, USA; pslath@gwu.edu; 5 Neuroscience Laboratory, The Felsenstein Medical Research Center, Sackler School of Medicine, Tel-Aviv University, Tel-Aviv 6997801, Israel; danioffen@gmail.com

**Keywords:** renal disease, DJ-1, ND-13, renal inflammation, oxidative stress, UUO, fibrosis

## Abstract

DJ-1 is a redox-sensitive chaperone with reported antioxidant and anti-inflammatory properties in the kidney. The 20 amino acid (aa) peptide ND-13 consists of 13 highly conserved aas from the DJ-1 sequence and a TAT-derived 7 aa sequence that helps in cell penetration. This study aimed to determine if ND-13 treatment prevents the renal damage and inflammation associated with unilateral ureter obstruction (UUO). Male C57Bl/6 and *DJ-1^−/−^* mice underwent UUO and were treated with ND-13 or vehicle for 14 days. ND-13 attenuated the renal expression of fibrotic markers *TGF-β* and *collagen1a1* (*Col1a1*) and inflammatory markers *TNF-α* and *IL-6* in C57Bl/6 mice. *DJ-1^−/−^* mice treated with ND-13 presented similar decreased expression of *TNF-α*, *IL-6* and *TGF-β*. However, in contrast to C57Bl/6 mice, ND-13 failed to prevent renal fibrosis or to ameliorate the expression of *Col1a1* in this genotype. Further, UUO led to elevated urinary levels of the proximal tubular injury marker neutrophil gelatinase-associated lipocalin (NGAL) in *DJ-1^−/−^* mice, which were blunted by ND-13. Our results suggest that ND-13 protects against UUO-induced renal injury, inflammation and fibrosis. These are all crucial mechanisms in the pathogenesis of kidney injury. Thus, ND-13 may be a new therapeutic approach to prevent renal diseases.

## 1. Introduction

Renal oxidative stress and inflammation are two of the most important factors involved in the pathogenesis of renal diseases and other cardiovascular disease complications [1]. Inflammation, the consequent oxidative stress, and vice versa, are considered major factors triggering fibrosis, and are key components in the development and progression of renal failure [2]. Renal fibrosis is caused, in part, by excess deposition of extracellular matrix, and inflammation is one of the main pathways that trigger this mechanism [3]. The inflammatory response during the initial stages of renal disease is characterized by glomerular and tubulo-interstitial infiltration of immune cells, including neutrophils and macrophages [4]. Furthermore, the activation of neutrophils during these early stages results in the release of proinflammatory and profibrogenic cytokines [4], followed by the infiltration of macrophages and T and B lymphocytes into the tissues. Macrophages are a major source of TGF-β in fibrotic organs [5,6], and recruitment of T and B lymphocytes to the site of injury further facilitates the secretion of fibrogenic cytokines [7,8]. TGF-β is also a potent chemoattractant involved in the recruitment of inflammatory cells [9] and, thereby, facilitates the expansion of the inflammatory process. Renal inflammation plays a central role in the initiation and progression of fibrosis in chronic kidney disease. Therefore, attenuation of the inflammatory response may be a critical step for the restoration of the proper balance between pro and antifibrotic signaling pathways [10,11] in the kidney and other organs.

DJ-1, also known as Park 7, is a multifunctional oxidative stress response protein. *DJ-1* was initially identified as an autosomal recessive gene associated with Parkinson’s disease, and it has been shown to be expressed in the brain, heart, kidney, liver, pancreas, and skeletal muscle in rodents as well as in humans [12]. DJ-1 functions as a redox-sensitive chaperone with intrinsic antioxidant properties, especially in the mitochondria, and it regulates the expression of several antioxidant genes such as glutathione and heat shock protein 70 in dopaminergic neurons [13,14]. DJ-1 is mainly present in the cytoplasm and, to a lesser extent, in the mitochondria. However, upon an oxidant challenge, DJ-1 translocates from the cytoplasm to the mitochondria where it protects mitochondrial function [15]. In a previous report, our group demonstrated that renal DJ-1 plays a critical role in the regulation of oxidative stress-dependent hypertension in mice [16].

Nrf2 (nuclear factor erythroid 2-related factor 2) is a transcription factor that regulates the expression of several antioxidant genes and also inhibits the development and progression of acute kidney injury caused by heavy metals, ischemia and xenobiotics such as cyclosporin A and cisplatin [17]. Nrf2 attenuates the NFκB-inflammatory pathway and suppresses proinflammatory cell signaling [18,19]. We previously reported that the kidney-selective silencing of *DJ-1* in mice leads to impairment of the antioxidant response mediated by the dopamine receptor 2 and increases in blood pressure associated with decreased Nrf2 expression and activity in the kidney [16,20]. In addition, mice with *DJ-1* selectively silenced in the kidney, and mice with germline deletion of *DJ-1* (*DJ-1^−/−^* mice), develop high blood pressure, renal damage and decreased kidney expression and activity of Nrf2 [20], suggesting that DJ-1 inhibits the production of renal reactive oxygen species (ROS), at least in part, via the activation of Nrf2-controlled antioxidant genes. We also recently demonstrated the important role of the antioxidant protein UCP2 in hypertension associated with the depletion of DJ-1 [21]. Moreover, other reports also implicated the DJ-1/Nrf2 pathway in the pathogenesis of several renal diseases, such as diabetic nephropathy in rats [22].

Dr. Daniel Offen’s laboratory, at the University of Tel Aviv, developed a 13 aa-long peptide derived from the most conserved sequence of DJ-1 [23]. To achieve cell permeability, this 13 aa chain was fused to a 7 aa TAT sequence (YGRKKRR). The resulting 20 amino acid compound was named ND-13 and was demonstrated to be effective in protecting neuronal cultures from the effects of relevant neurotoxins in the setting of Parkinson’s disease, amyotrophic lateral sclerosis or multiple system atrophy [23,24,25]. ND-13 exerts these protective effects by reducing apoptosis and by inactivating the proapoptotic protein caspase-3 in neuronal cell lines exposed to these neurotoxic insults. In those studies, ND-13 treatment led to the activation of the Nrf2 pathway and the consequent increased expression of Nrf2-induced antioxidant genes [23], similar observations to our previous findings in the kidney of *DJ-1*^−/−^ mice [20]. However, the potential protective effects of ND-13 against the development of renal disease remain unknown. Therefore, the goal of these studies was to determine if ND-13 prevents the renal damage and inflammation associated with an animal model of progressive kidney fibrosis, the unilateral ureter obstruction (UUO) model, and if this protection is mediated by the activation of the DJ-1/Nrf2 pathway.

## 2. Results

### 2.1. ND-13 Treatment Reduces UUO-Induced Renal Fibrosis in WT Mice, but Not in DJ-1^−/−^ Mice

**Figure 1:**
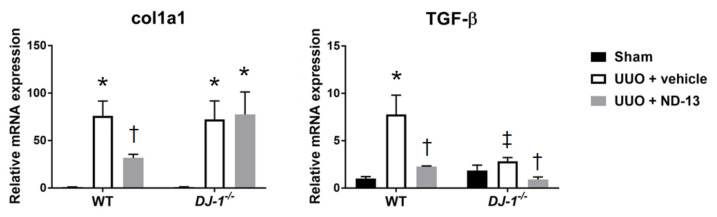
Treatment with ND-13 reduces the unilateral ureter obstruction (UUO)-induced renal expression of fibrotic markers in wild type (WT) mice, but not in *DJ-1*^−/−^ mice. Relative mRNA expression of markers of fibrosis *col1a1* and *TGF-β* in renal cortex of WT and *DJ-1*^−/−^ mice that underwent sham surgery, UUO and vehicle treatment or UUO and ND-13 treatment. *N* = 4–5/group; * *p* < 0.05 vs. same genotype sham, † *p* < 0.05 vs. same genotype UUO + vehicle, ‡ *p* < 0.05 vs. WT UUO + vehicle; two-way ANOVA with Tukey’s post-hoc test.

Because UUO is a classical model of progressive renal fibrosis, we evaluated by RT-PCR the expression levels of markers of fibrosis in the cortex of wild type (WT) mice that underwent sham or UUO surgery and were treated with ND-13 or vehicle for 14 days. We found that after UUO surgery, WT mice treated with vehicle presented a significant upregulation of the markers of fibrosis *Col1a1* and *TGF-β* (~70-fold increase for *Col1α1* and ~7-fold increase for *TGF-β p* < 0.05; Figure 1) in the renal cortex. Importantly, daily treatment with ND-13 significantly decreased the cortical expression of both markers of fibrosis, suggesting a protective effect of this peptide against the development of the cortical fibrosis typically induced by UUO.

In order to characterize the role of DJ-1 in the development of the renal fibrosis associated with UUO, we performed similar studies using the *DJ-1* global knockout (*DJ-1*^−/−^) mouse. In response to UUO, and similar to our findings in the WT mice, *DJ-1*^−/−^ mice significantly upregulated the mRNA expression of the marker of fibrosis *Col1a1* compared to the levels that the sham group presented (Figure 1). However, and contrary to the results found in WT mice, treatment of *DJ-1*^−/−^ mice with ND-13 failed to prevent the elevation of *Col1a1* in the cortex after UUO. In contrast, the cortical levels of *TGF-β* after UUO did not significantly increase in the *DJ-1*^−/−^ mice compared to sham controls, but interestingly, treatment with ND-13 was efficient in decreasing the mRNA expression of *TGF-β* in the renal cortex (Figure 1).

**Figure 2:**
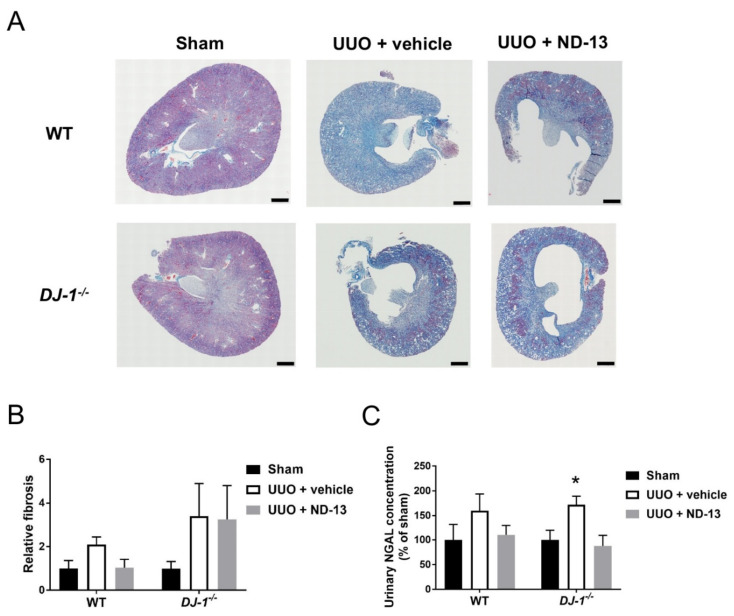
Treatment with ND-13 reduces UUO-induced renal fibrosis in WT mice. (**A**) Representative full scan images of Masson’s blue trichrome-stained kidneys obtained from WT and *DJ-1*^−/−^ mice that underwent sham surgery, UUO and vehicle treatment or UUO and ND-13 treatment (scale bar = 500 μm). (**B**) Quantification of collagen deposition in renal cortex of WT and *DJ-1*^−/−^ mice that underwent sham surgery, UUO and vehicle treatment or UUO and ND-13 treatment. (**C**) Urinary concentration of the proximal tubule marker NGAL in WT and *DJ-1*^−/−^ mice that underwent sham surgery, UUO and vehicle treatment or UUO and ND-13 treatment. *n* = 4–5/group; * *p* < 0.05 vs. same genotype sham, two-way ANOVA with Tukey’s post hoc test.

**Figure 3:**
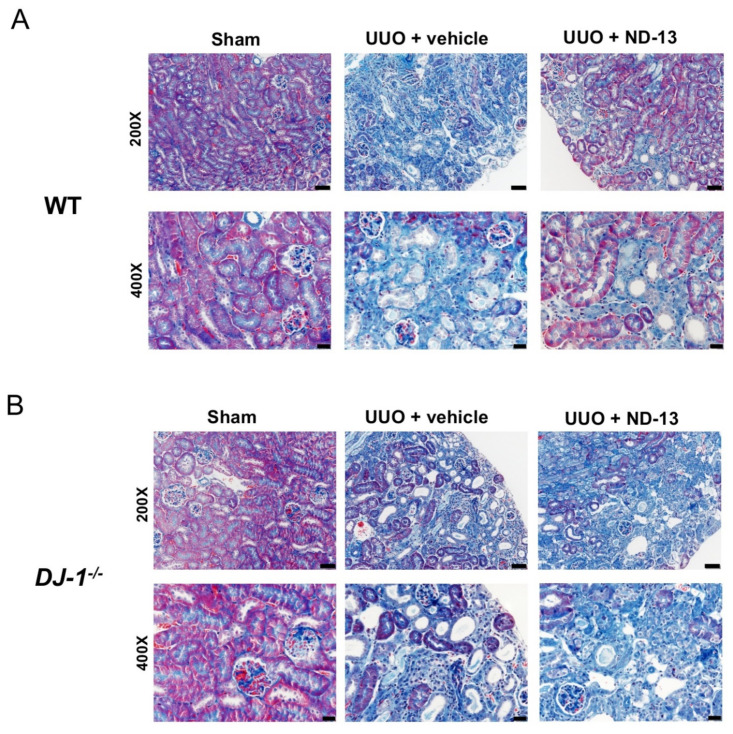
Treatment with ND-13 seems to blunt the spread of UUO-induced fibrosis accumulation in the cortex of WT mice but fails to do so in *DJ-1*^−/−^ mice. (**A**) Representative Masson’s blue trichrome images of renal cortex of WT mice that underwent sham surgery, UUO and vehicle treatment or UUO and ND-13 treatment: 200× (upper panels; scale bar = 50 μm) and 400× magnification (bottom panels; scale bar = 20 μm). (**B**) Representative Masson’s blue trichrome images of renal cortex of *DJ-1*^−/−^ mice that underwent sham surgery, UUO and vehicle treatment or UUO and ND-13 treatment: 200× (upper panels; scale bar = 50 μm) and 400× magnification (bottom panels; scale bar = 20 μm).

Similar to the results obtained by RT-PCR, Masson’s blue trichrome histological staining demonstrated extensive interstitial fibrosis in the cortex, outer and inner medulla in WT mice treated with vehicle, as indicated by the homogeneous presence of blue staining in Figure 2A. Although deposition of fibrotic material was also apparent in the WT mice treated with ND-13, examination of the tissue at higher magnification revealed that the cortical fibrosis was not as homogenously distributed, and tended to be localized to certain areas of the cortex while other areas appeared fibrosis-free (Figure 2A and Figure 3A), suggesting that treatment with ND-13 may be blunting the accumulation of fibrotic material in the renal cortex. Despite these observations of the extension of fibrosis and the clear trend of the results, no significant difference in the cortical fibrosis quantification was found among the WT groups (relative fibrosis: sham: 1 ± 0.4, UUO + vehicle: 2.1 ± 0.3 and UUO + ND-13: 1.0 ± 0.4, *p* > 0.05, *n* = 4–5/group, Figure 2B).

Likewise, histological evaluation of cortical tissue obtained from *DJ-1*^−/−^ mice after the UUO protocol supported the molecular findings in this genotype, as ND-13 treatment had no effect on the amount of collagen deposition detected by trichrome blue staining in this region of the kidney (relative fibrosis, UUO + vehicle vs. UUO + ND-13: 3.4 ± 1.5 vs. 3.3 ± 1.5; Figure 2A,B and Figure 3B). The renal fibrosis in *DJ-1*^−/−^ mice was found to be evenly distributed across the cortical region (Figure 3B).

To determine the extent of renal damage, the concentration of neutrophil gelatinase-associated lipocalin (NGAL), a marker of proximal tubule damage, was measured in urine. We found that the urinary concentrations of NGAL were similar among the three experimental groups of WT mice, suggesting that these groups presented similar levels of renal damage (Figure 2C). On the other hand, fourteen days after UUO, *DJ-1*^−/−^ mice presented significantly greater amounts of urinary NGAL compared to *DJ-1*^−/−^ mice that underwent sham surgery, and daily treatment of these mice with ND-13 resulted in a normalization of these urinary values to sham levels (Figure 2C). These results suggest that *DJ-1*^−/−^ mice are more sensitive to UUO-induced kidney damage, and that ND-13 ameliorates the damage inflicted to the proximal tubules by UUO.

### 2.2. Treatment with ND-13 Does Not Prevent UUO-Induced Cell Death in the Kidney

**Figure 4:**
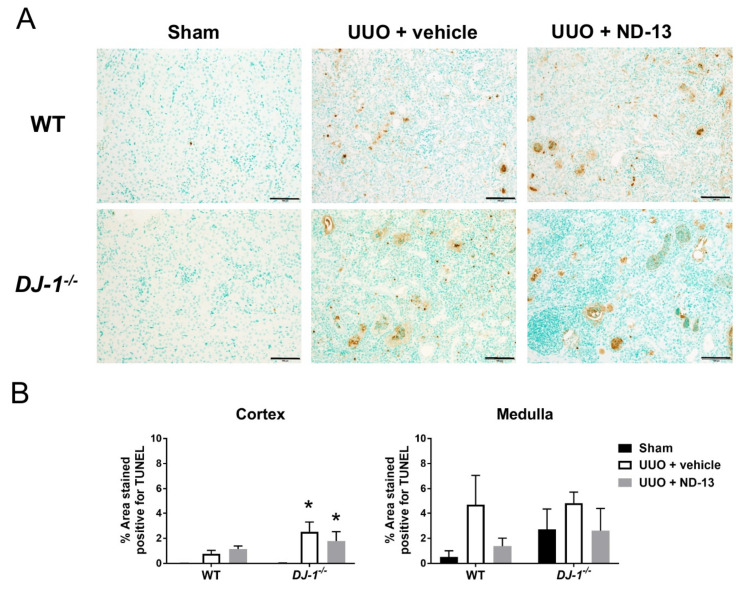
ND-13 does not protect against UUO-induced cell death. (**A**) Representative images of TUNEL stained (brown color) kidney cortex from WT (upper panels) and *DJ-1*^−/−^ mice that underwent sham surgery, UUO and vehicle treatment or UUO and ND-13 treatment (scale bar = 100 μm). (**B**) Quantification of cell death (% TUNEL-positive area) in kidney cortex and medulla of WT and *DJ-1*^−/−^ mice that underwent sham surgery, UUO and vehicle treatment or UUO and ND-13 treatment. *n* = 4–5/group; * *p* < 0.05 vs. same genotype sham, two-way ANOVA with Tukey’s post-hoc test.

To determine if treatment with ND-13 effectively protected against kidney cell death in response to the UUO protocol, the presence of Terminal Deoxynucleotidyl Transferase-Mediated dUTP Nick-End Labeling (TUNEL)-positive areas in kidney cortex and medulla was quantified in both genotypes. As shown in Figure 4A, and as expected, WT and *DJ-1^−/−^* sham animals hardly presented any TUNEL-positive cells in the kidney cortex. UUO induced cell death in mice of both genotypes treated with vehicle, although the elevation in the percentage area of the kidney that stained positive for TUNEL only reached significance in the *DJ-1^−/−^* mice, and appeared to be greater than in WT mice (*p* > 0.05, Figure 4B). Upon closer examination, the cortical TUNEL-positive stain was observed in glomeruli, tubular cells and interstitial cells in both genotypes. Treatment with ND-13 was unable to prevent cortical cell death in either genotype, as the percentage area of the kidney stained for TUNEL remained elevated in both. No differences between the treatments and genotypes were observed in the renal medulla (Figure 4B).

### 2.3. Renal Expression of Cytokine and Chemokine Genes Associated with UUO is Ameliorated in Mice Treated with ND-13

**Figure 5:**
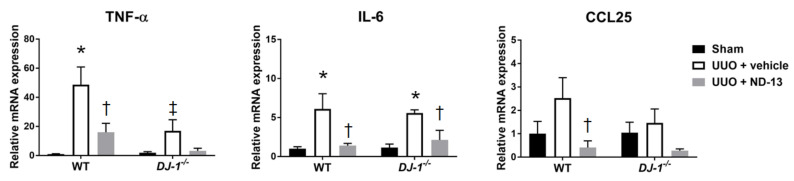
Expression of cytokine and chemokine genes associated with UUO is attenuated in kidneys obtained from WT mice treated with ND-13. Relative mRNA expression of cytokines *TNF-α* and *IL-6* and chemokine *CCL25* in renal cortex of WT mice and *DJ-1^−/−^* mice that underwent sham surgery, UUO and vehicle treatment or UUO and ND-13 treatment. *n* = 4–5/group; * *p* < 0.05 vs. same genotype sham, † *p* < 0.05 vs. same genotype UUO + vehicle, ‡ *p* < 0.05 vs. WT UUO + vehicle; two-way ANOVA with Tukey’s post-hoc test.

Renal inflammation has been proven to be intimately associated with the development of kidney damage [2]. Therefore, we evaluated the level of kidney inflammation in our experimental animals and found that WT mice that underwent UUO, and were treated with vehicle, had significantly upregulated expression of cytokines *TNF-α* and *IL-6* and chemokine *CCL25* in the renal cortex (Figure 5). Interestingly, daily treatment with ND-13 led to a significant attenuation in the expression of these markers of inflammation, bringing their expression levels to values similar to those found in the sham WT group.

Similar to the trends found in WT mice, ND-13 had anti-inflammatory effects on *DJ-1*^−/−^ mice that underwent UUO surgery, as indicated by the blunted expression of *TNF-α*, *CCL25* and *IL-6* in the renal cortex compared to the values observed in the vehicle-treated group (Figure 5). Of note, the *TNF-α* response to UUO was significantly smaller in *DJ-1^−/−^* mice treated with vehicle than the response observed in WT mice treated with vehicle (Figure 5).

### 2.4. ND-13 Treatment Attenuates the UUO-Induced Cortical Macrophage Inflammation in WT Mice, but not in *DJ-1^−/−^* Mice, While not Preventing the Infiltration of T-Lymphocytes in Either Genotype

**Figure 6:**
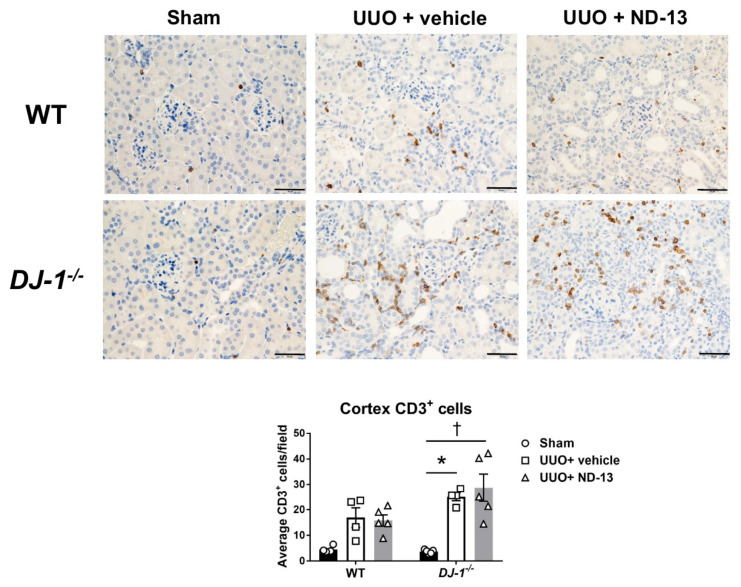
Treatment with ND-13 does not prevent the UUO-induced infiltration of T cells into the kidney cortex of mice that underwent UUO. Representative images and quantification of T cell (CD3^+^ cells) infiltration in renal cortex of WT (upper panels) and *DJ-1^−/−^* (bottom panels) mice that underwent sham surgery, UUO and vehicle treatment or UUO and ND-13 treatment (scale bar = 20 μm). * *p* < 0.05 vs. same genotype sham, † *p* < 0.05 vs. same genotype UUO + vehicle; two-way ANOVA with Tukey’s post hoc test.

As expected, the population of T-lymphocytes (CD3^+^ cells) in the cortex was elevated in WT mice after UUO, although it did not reach statistical significance from the levels shown in mice that underwent sham surgery (sham vs. UUO + vehicle: 4.6 ± 0.7 vs. 14.9 ± 4.6 cells/field, *p* > 0.05, *n* = 4–5/group). Daily treatment with ND-13 failed to normalize the kidney T-cell inflammation in WT mice (16.1 ± 2.8 cells/field; Figure 6). Similarly, *DJ-1^−^*^/^*^−^* mice showed an increased infiltration of T-lymphocytes in the renal cortex (Figure 6) in response to UUO (sham vs. UUO + vehicle: 3.7 ± 0.3 vs. 24.9 ± 2.2 cells/field, *p* < 0.05, *n* = 4–5/group). However, the magnitude of T cell accumulation in the cortex of *DJ-1^−^*^/^*^−^* mice was greater than in WT mice (WT mice vs. *DJ-1^−^*^/^*^−^* mice: 14.9 ± 4.6 vs. 24.9 ± 2.2 cells/field), and it remained elevated despite the ND-13 treatment (26.0 ± 8.3 cells/field). Raw data for the T cell numbers in each of the 10 fields evaluated per animal, as well as the mean and SEM per animal, are provided in Supplementary Table S1.

**Figure 7:**
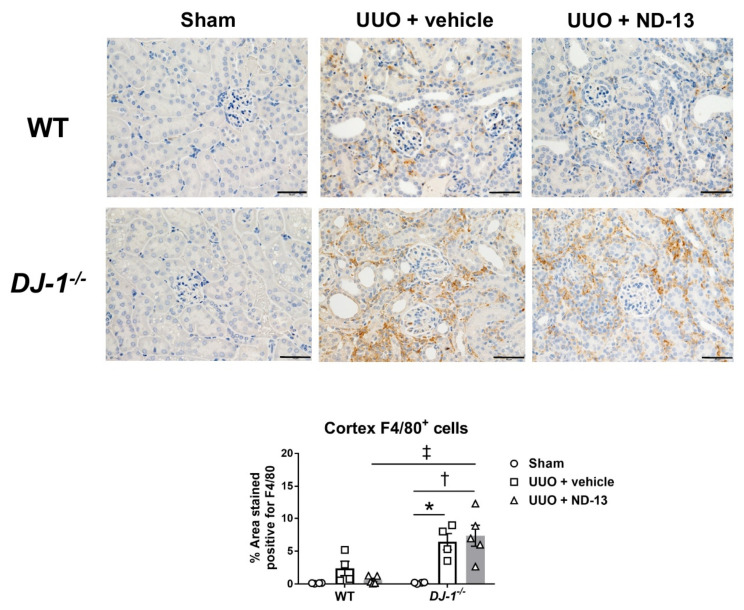
Treatment with ND-13 tends to decrease the UUO-induced macrophage infiltration into the renal cortex of WT mice that underwent UUO but fails to prevent this inflammation in *DJ-1^−/−^* mice that underwent UUO. Representative images and quantification of macrophage infiltration (% area stained for F4/80) in renal cortex of WT mice that underwent sham surgery, UUO and vehicle treatment or UUO and ND-13 treatment (scale bar = 20 μm). * *p* < 0.05 vs. same genotype sham, † *p* < 0.05 vs. same genotype UUO + vehicle, ‡ *p* < 0.05 vs. WT UUO + vehicle; two-way ANOVA with Tukey’s post hoc test.

Similar to what was found with T cells, the number of macrophages infiltrating the cortex was elevated fourteen days after UUO surgery (sham vs. UUO + vehicle: 0.1 ± 0.0 vs. 2.4 ± 1.1 % area stained positive for F4/80, *p* > 0.05, *n* = 4–5/group). However, treatment with ND-13 only tended to attenuate those numbers (0.7 ± 0.3 % area stained positive; Figure 7). Evaluation of the macrophage population in the renal cortex of *DJ-1^−/−^* mice also revealed elevated numbers of these immune cells after UUO (sham vs. UUO + vehicle: 0.2 ± 0.1 vs. 6.5 ± 1.2 % area stained positive for F4/80, *p* < 0.05, *n* = 4–5/group); however, in contrast to WT mice, treatment with ND-13 failed to prevent macrophage infiltration in these mice, and the numbers of macrophages were similar to those found in mice treated with vehicle (7.0 ± 2.0 % area stained positive) (Figure 7). Of note, the magnitude of the cortical infiltration of macrophages after UUO was significantly worse in the case of the DJ-1*^−^*^/^*^−^* mice compared to WT mice (UUO + vehicle, WT mice vs. *DJ-1^−^*^/^*^−^* mice: 2.4 ± 1.1 vs. 6.5 ± 1.2 % area stained positive, *p* < 0.05, *n* = 4–5/group). Raw data for the percentage area stained positive for F4/80 quantified in each of the 10 fields/animal, as well as the mean and SEM per animal, are provided in Supplementary Table S2.

### 2.5. ND-13 Does Not Improve the Renal Damage Score in UUO Mice

The tubular damage and neutrophilic infiltrates present in the kidneys obtained from the experimental mice were evaluated using hematoxylin and eosin (H&E) stained sections and given a combined renal damage score. Kidneys obtained from UUO mice showed epithelial flattening and focally dilated tubules, with moderate proteinaceous contents and casts, and showed no difference between genetic backgrounds or treatment conditions. The glomerular morphology presented unremarkable changes in all groups. The renal damage score based on H&E staining showed a very small, insignificant protective effect of ND-13 on the extent of the neutrophilic infiltrate in the cortex. The means and standard errors of these inflammation scores, adjusted by density, are the following: C57BL/6 mice with UU0 + vehicle: 1.45 ± 0.83 vs. UU0 + N-13: 1.15 ± 0.81; *DJ-1^−^*^/^*^−^* mice with UU0 + vehicle: 3.13 ± 1.04 vs UU0 + N-13: 2.31 ± 1.14. Significant differences were not found between the groups. No inflammation was found in the control mice and no apparent morphological changes were found in the contralateral kidneys.

## 3. Discussion

The major finding of this study is that treatment with ND-13 is effective in preventing the exaggerated expression of kidney fibrotic and inflammatory markers that normally develop as consequences of UUO. We demonstrated that ND-13 significantly reduced the expression of fibrotic markers col1a1 and TGF-β and prevented the accumulation of macrophages in the kidney of C57Bl/6J mice after UUO. The fact that ND-13 did not protect *DJ-1^−/^^−^* mice against the UUO-induced renal damage highlights the critical role of DJ-1 as an important mediator of this protective mechanism. To our knowledge, this is the first report to evaluate the protective effects of ND-13 in renal diseases.

According to the Center for Disease Control, 37 million Americans suffered from chronic kidney disease in 2019, with associated health costs of about $114 billion to care for these patients [26]. Current therapies are not effective in preventing the development of renal disease [27]. Thus, new therapeutic alternatives are urgently needed. There is abundant evidence in the literature that demonstrates that renal inflammation and renal fibrosis precede the development of chronic kidney disease [28], highlighting the critical involvement of these two factors in the pathogenesis of renal damage. Among other kidney diseases, inflammation and fibrosis are involved in the progression of glomerulonephritis [29], acute kidney injury [7], polycystic kidney disease [30], renal artery stenosis [31], lupus nephritis [32] and diabetic nephropathy [33]. Accordingly, pharmacological therapies aimed at the attenuation of inflammatory and fibrotic processes may be an appropriate approach in the prevention of these renal pathologies.

UUO is a model of progressive kidney fibrosis and inflammation that is characterized by tubular dilation, loss of proximal tubular mass, interstitial expansion, hypertrophy, hydronephrosis and tubular epithelial cell death [34]. The mechanical stretching induced by the tying of the ureter stimulates a massive production of reactive oxygen species (ROS) and cellular apoptosis in the affected kidney that, in turn, result in alterations of the hemodynamic status and significant inflammation and fibrosis [34]. Therefore, UUO is an ideal experimental model to evaluate the putative preventive effects of ND-13 against renal damage and, particularly, on kidney fibrosis.

To examine DJ-1 as a possible novel therapeutic target for renal diseases, we used the 20 aa peptide known as ND-13 [23]. Treatment of WT mice with ND-13 blunted the UUO-induced upregulation in the kidney expression of *TGF-β* and *Col1a1*, and also decreased the kidney expression of inflammatory markers *TNF-α*, *IL-6* and *CCL25*. Interestingly, the ND-13-mediated decrease in fibrotic marker expression was not accompanied by a significant improvement in the deposition of collagen in the kidney. These seemingly contradictory findings could be due to the long timeline in our studies, where the animals were examined a full two weeks after the UUO protocol. This timeline, compared to shorter postsurgical times, is known to induce severe injury to the kidney and stimulate extreme deposition of collagen in this organ [34,35,36]. The protective effects of ND-13 against collagen deposition may have been hindered by starting the treatment at the same time as the UUO pathology. Interestingly, and although it did not reach statistical significance, we observed that the cortical fibrosis in the WT mice treated with ND-13 was not as generalized as it was in mice treated with vehicle, possibly suggesting that treatment with ND-13 may have slightly slowed down the fibrotic deposition. Considering the profound reduction in kidney fibrotic marker expression induced by ND-13, it is likely that its effects on kidney fibrosis would have been different if the animals were treated with this peptide for a period of time prior to the UUO protocol. This is a research avenue that our group will investigate in the future. Similarly, the protective effects of ND-13 did not extend to the renal T cell infiltration, which remained elevated. However, it seemed to prevent the macrophage influx into this organ. It is possible that ND-13 shifted the phenotype of the T cell and macrophage populations present in the kidney from a proinflammatory type to an anti-inflammatory type (i.e., Tregs vs. Th17 cells, or M1 vs. M2 macrophages) attenuating the inflammatory response and renal damage. Future follow-up studies will focus on the further evaluation of immune cell subtypes present in the kidneys of these animals in order to completely understand the effects of ND-13 in proinflammatory versus anti-inflammatory immune populations in the kidney after UUO. Moreover, these results suggest promising protective effects of ND-13 that could be more evident in less aggressive animal models of kidney disease with pathogenic mechanisms more similar to human renal disease.

On the other hand, and despite the attenuation in the expression of inflammatory cytokines that we observed in *DJ-1^−/−^* mice that underwent UUO and were treated with ND-13, we did not find differences in kidney fibrosis nor collagen deposition in these mice when compared with the shams. The fact that treatment with ND-13 did not decrease kidney fibrosis in *DJ-1^−/−^* mice, but it did in WT mice, strongly underlines the essential role of renal DJ-1 in the prevention of renal damage.

Our group previously reported the effects that deletion of *DJ-1* has on renal oxidative stress and injury as well as on blood pressure [16,20,21] and a protective role of DJ-1 in endotoxin-induced acute kidney injury was recently described [36]. We demonstrated that silencing *DJ-1* expression in mouse kidneys, and in mouse proximal tubule cells specifically, attenuates the expression and activity of Nrf2 and results in increased ROS production [20]. Nrf2 is a master regulator of antioxidant and anti-inflammatory factors [19], and its activity and expression is regulated by ROS production [37]. Moreover, genetic deletion of *DJ-1* leads to increased ubiquitination of Nrf2, suggesting that the renal protection exerted by DJ-1 is mediated by preventing the degradation of Nrf2 [20]. Interestingly, both DJ-1 and Nrf2 are activated in acute kidney injury [38], and Nrf2 has been proven to inhibit the development and progression of several diseases affecting the kidney [17,39]. In previous studies, we also demonstrated that DJ-1 increases Nrf2 expression and activity only under pathological conditions and has no effects on Nrf2 in the physiological setting.

**Figure 8:**
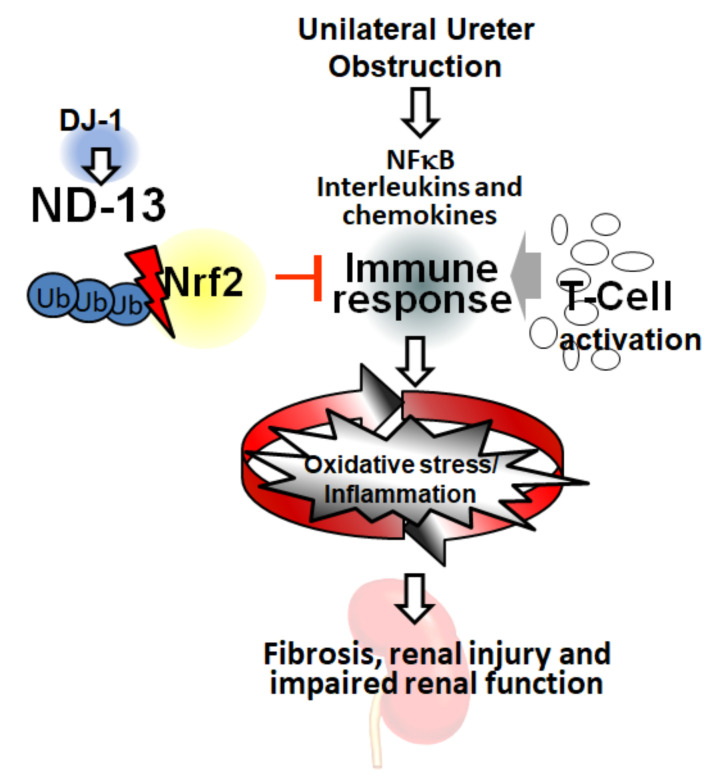
Working hypothesis. ROS and inflammation induced by unilateral ureter obstruction increase interleukin and chemokine expression activating the immune response. This inflammatory/oxidative environment leads to a vicious cycle, which may produce fibrosis and induce renal damage and kidney dysfunction. DJ-1 prevents the ubiquitination of Nrf2 and amplifies its response. Nrf2 may be acting by attenuating the oxidative stress and inflammation, leading to a reduced cytokine expression and preventing inflammation, resulting in protection of the renal function.

Consistent with our results, previous studies demonstrated that DJ-1 stabilized Nrf2 by preventing binding to Keap1 and Nrf2’s subsequent ubiquitination [40]. DJ-1 may amplify Nrf2 activity by avoiding its degradation. However, the ability of DJ-1 to stimulate directly the Nrf2 pathway has not been demonstrated [41]. We speculate that ND-13 prevents the undesirable consequences of chronic Nrf2 activation. Thereby, ND-13 may be an appropriate therapeutic approach to enhance the actions of Nrf2, and it could be used to minimize the side effects associated with treatment of chronic Nrf2 activation by other Nrf2 inducers i.e., bardoxolone [42,43] in humans. Our working hypothesis is that the ROS and inflammation that are induced by UUO lead to increased levels of interleukins and chemokines and, in turn, to the activation of the immune response in the kidney. This inflammatory and oxidative milieu creates a vicious cycle that leads to fibrosis and promotes kidney damage and kidney dysfunction. In this setting, DJ-1 prevents the ubiquitination of Nrf2, amplifying this molecule’s antioxidant response. We speculate that Nrf2 would attenuate oxidative stress and inflammation in the kidney, leading to reduced cytokine expression and thereby preventing inflammation and preserving renal function (Figure 8).

In summary, these data suggest that ND-13 prevents the renal inflammation and fibrosis associated with the acute renal damage induced by UUO. Further studies are needed to confirm if ND-13 treatment may be a new therapeutic approach for the prevention of renal injury, fibrosis and inflammation in human renal disease.

## 4. Materials and Methods

### 4.1. Animal Studies

All protocols were conducted in accordance with the Guide for the Care and Use of Laboratory Animals, and were approved by the Institutional Animal Care and Use Committee of the George Washington University (project identification numbers A353 and A412, approved on 26 February 2019 and 21 February 2019, respectively). Eight to nine-week old male C57Bl/6J (Jax Labs, Bar Harbor, ME) or *DJ-1^−/−^* mice (from our in-house colony) were used in these studies. Mice of each genotype underwent sham or unilateral ureter obstruction (UUO) surgery. In short, mice were anesthetized, the lower abdomen was opened and the left ureter was completely tied off using suture [44]. In those mice in the sham group, the abdomen was opened, the left ureter touched with a cotton tipped applicator and the abdomen was then sutured. The mice that underwent UUO surgery received either ND-13 (3 mg/kg/day, s.c.) or vehicle (scrambled peptide; 3 mg/kg/day, s.c.) starting from the day after surgery until the end of the study. Twelve days into the study, mice were placed in metabolic cages, acclimated to the cages for one day, and 24 h-urine was collected on the second day on the metabolic cages. On day 14 of the study, the mice were sacrificed, and kidneys harvested. One of the kidneys was snap-frozen in liquid nitrogen, while the other was placed in formalin for histological studies. No significant differences were found among the experimental groups regarding body weight, food intake, water intake or urine production (Table 1).

### 4.2. Quantitative RT-PCR

RNeasy mini kit (Qiagen, Valencia, CA, USA) was used to extract RNA from the kidney cortex. The amount of extracted RNA was quantified by spectrophotometry (NanoDrop ND-1000, Thermo Scientific, Waltham, MA, USA). RNA reverse transcription was performed using Quantitect Reverse Transcription kit (Qiagen) and following manufacturer’s instructions. Primers were purchased from QuantiTect (Qiagen): *Col1a1* (QT00371308), *TGF-β* QT00371308), *TNF-α*QT00371308), *CCL25* (QT00371308) and *IL-6* (QT00371308). GAPDH was used as housekeeping gene (QT01658692). RNA expression was detected by the Quantitect SYBR green kit (Qiagen) and using a CFX96 Touch RT-PCR detection system (Bio-Rad, Hercules, CA, USA).

### 4.3. Histology and Fibrosis Quantification

Kidneys were fixed overnight in 4% buffered formalin solution at room temperature, transferred to 70% ethanol for 24 h, and paraffin-embedded. Tissues were cut longitudinally into 4 μm-thick sections and mounted on Superfrost slides. Masson’s trichrome blue staining was used to visualize renal fibrosis using bright-field microscopy (Olympus BX40 with 10× eyepiece lens; Olympus America, Melville, NY, USA). Full scans of the kidneys were obtained using a microscope fitted with a motorized XY stage and a digital camera (Olympus DP71), with sequential 20× images of each kidney taken and digitally stitched together with CellSense imaging software (Olympus). The cortical area of each full kidney scan was outlined and the percentage of blue fibrotic deposition within the outlined area was quantified using MetaMorph software (Molecular Devices LLC., San Jose, CA, USA). The average percentage fibrotic area for each experimental group was calculated and then normalized to the sham group. Data are presented as relative fibrosis compared to sham group.

### 4.4. Immunohistochemistry and Quantification of Immune Cell Infiltration in the Kidney

Tissue sections were stained with primary antibodies specific for CD3 (1:600; Abcam, Cambridge, MA, USA) and F4/80 (1:200; Bio-Rad, Hercules, CA, USA) and detected with polymer conjugated secondary antibody (Biocare Medical, Concord, CA, USA). Quantification of renal T-lymphocyte (CD3^+^ cells) infiltration was performed by blindly counting 10 microscopic fields (200 × 200 μm, 400× magnification) in each renal cortex. Infiltrating T cell numbers are reported as the average of the counts in the 10 fields per renal cortex. Quantification of renal macrophages (F4/80^+^ cells) was performed by taking 10 cortical images from each animal at 400× magnification. The percentage of cortical area stained positive for F4/80 was quantified in each image using MetaMorph software and the average expression per animal was calculated. The data are reported as the average percentage area that stained positively for F4/80 per experimental group.

### 4.5. Terminal Deoxynucleotidyl Transferase-Mediated dUTP Nick-End Labeling (TUNEL) Assay and Quantification

Tissue sections were stained using the Apoptag^®^ Plus Peroxidase In Situ Apoptosis Kit (S7101, MP Biomedicals, Santa Ana, CA, USA) in order to detect dead cells. Ten microscopy images were taken of each kidney cortex and medulla (400 × 400 μm fields at 200× magnification) and the area stained positively with TUNEL in each image was quantified using Metamorph software and averaged per kidney region and animal. The data are reported as the average percentage area that stained positive for TUNEL in cortex and medulla per experimental group.

### 4.6. Urinary NGAL Measurements

Urine collected on day two of the metabolic cage study was analyzed for concentration levels of the proximal tubular injury marker neutrophil gelatinase-associated lipocalin (NGAL) using an ELISA kit (Abcam). Average concentration per experiment group was calculated and the data are presented as percentage of sham group.

### 4.7. Renal Damage Evaluation

Five images per mouse kidney section stained with H&E were evaluated by a blinded pathologist and each mouse was assigned a renal damage score based on the following criteria: inflammatory involvement (1 = <25%, 2 = 25–50%, 3 = 50–75%, 4 = >75%), inflammatory density (1 = mild, 2 = mild- moderate, 3 = moderate-severe, 4 = severe), dilation of tubules and tubular injury with neutrophilic infiltrates (PMNs) (1 = <2, 2 = 2–4, 3 = several, 4 = many) and casts (1 = <3, 2 = easily found; 3 = many).

### 4.8. Statistical Analysis

All data are expressed as mean ± SEM. Differences between groups were analyzed by two-way analysis of variance with a Tukey’s post hoc test. A *p* < 0.05 was considered statistically significant. All statistical analyses were conducted using SigmaPlot 11 (Systat Software, Inc., San Jose, CA, USA).

## Figures and Tables

**Table 1:** Physical characteristics of the experimental groups.

	Body Weight (g)					
	Pre		Post		Gain/Loss	
	Mean	SEM	Mean	SEM	Mean	SEM
WT sham	28.75	3.22	28.85	2.90	0.10	0.38
DJ-1*^−^*^/*−*^ sham	25.38	1.58	25.74	1.62	0.36	0.09
WT vehicle	23.30	2.05	23.94	2.00	0.64	0.31
DJ-1*^−^*^/*−*^ vehicle	26.20	3.12	26.40	3.18	0.20	0.16
WT ND-13	28.72	2.89	29.26	2.76	0.54	0.76
DJ-1*^−^*^/*−*^ ND-13	24.80	3.40	25.02	4.01	0.22	0.34
	**Food Intake (g)**					
	**Pre**		**Post**		**Net**	
	**Mean**	**SEM**	**Mean**	**SEM**	**Mean**	**SEM**
WT sham	75.85	0.57	72.40	2.95	3.45	2.57
DJ-1*^−^*^/*−*^ sham	74.00	3.74	70.84	2.99	3.16	1.14
WT vehicle	70.66	3.89	64.80	3.87	5.86	1.88
DJ-1*^−^*^/*−*^ vehicle	74.50	4.08	71.70	4.16	2.80	0.42
WT ND-13	71.06	3.14	65.56	3.76	5.50	1.29
DJ-1*^−^*^/*−*^ ND-13	71.78	3.02	68.53	3.21	3.25	1.41
	**Water Intake (mL)**					
	**Pre**		**Post**		**Net**	
	**Mean**	**SEM**	**Mean**	**SEM**	**Mean**	**SEM**
WT sham	53.43	6.34	50.73	7.44	2.70	1.31
DJ-1*^−^*^/*−*^ sham	55.44	4.97	51.48	3.90	3.96	1.60
WT vehicle	55.08	2.22	49.98	3.89	5.10	1.82
DJ-1*^−^*^/*−*^ vehicle	57.90	6.12	54.25	6.02	3.65	0.53
WT ND-13	56.08	1.93	50.34	2.28	5.74	0.57
DJ-1*^−^*^/*−*^ ND-13	59.58	2.75	55.45	2.22	4.13	1.97
	**Urine (mL)**					
			**Mean**	**SEM**		
WT sham			0.63	0.28		
DJ-1*^−^*^/*−*^ sham			0.69	0.32		
WT vehicle			1.10	0.36		
DJ-1*^−^*^/*−*^ vehicle			1.18	0.28		
WT ND-13			1.86	0.65		
DJ-1*^−^*^/*−*^ ND-13			1.38	0.51		

M: mean; SEM: standard error of the mean.

## Supplementary Materials

The following are available online at https://www.mdpi.com/1422-0067/21/19/7048/s1.

## Abbreviations

UUO	Unilateral ureter obstruction
Col1a1	Collagen 1a1
Nrf2	Nuclear factor erythroid 2-related factor 2
