# Supplementary material for: ND-13, a DJ-1-Derived Peptide, Attenuates the Renal Expression of Fibrotic and Inflammatory Markers Associated with Unilateral Ureter Obstruction
Source: Int J Mol Sci. 2020 Sep 24;21(19):7048. doi: 10.3390/ijms21197048 (PMC7582723; doi:10.3390/ijms21197048)
Supplement: Supplementary file 1 [file ijms-21-07048-s001.pdf]

Article

ND-13, a DJ-1-Derived Peptide, Attenuates the Renal Expression of Fibrotic and Inflammatory Markers Associated with Unilateral Ureter Obstruction

Table 1. Individual CD3+ cell counts per microscopy field.

Cortex CD3 stain

Slide Label	Treatment	Field 1	Field 2	Field 3	Field 4	Field 5	Field 6	Field 7	Field 8	Field 9	Field 10	Avg # per Field	SEM	n
1846/C1	C57Bl/6 control	4	6	2	2	0	3	6	5	7	4	3.9	0.690411	10
1847/C2	C57Bl/6 control	12	6	6	3	10	6	6	7	8	1	6.5	0.991632	10
1848/C3	C57Bl/6 control	3	3	3	2	0	0	7	6	9	4	3.7	0.919541	10
1849/C4	C57Bl/6 control	8	4	4	3	5	3	3	3	4	5	4.2	0.489898	10
1841R/C6	C57Bl/6 UUO + vehicle	8	5	7	25	6	8	13	1	1	4	7.8	2.2151	10
1842R/C7	C57Bl/6 UUO + vehicle	42	5	12	22	33	11	14	40	45	12	23.6	4.74037	10
1843R/C8	C57Bl/6 UUO + vehicle	40	10	10	20	30	7	13	37	46	18	23.1	4.460817	10
1844R/C9	C57Bl/6 UUO + vehicle	26	13	15	25	10	9	14	3	10	8	13.3	2.3	10
1836/C11	C57Bl/6 UUO + ND-13	14	13	9	23	7	17	26	7	12	20	14.8	2.085932	10
1837R/C12	C57Bl/6 UUO + ND-13	19	27	24	14	7	17	12	30	11	55	21.6	4.377214	10
1838R/C13	C57Bl/6 UUO + ND-13	17	12	10	15	9	13	21	20	6	25	14.8	1.884439	10
1839R/C14	C57Bl/6 UUO + ND-13	45	0	6	24	21	22	8	25	25	15	19.1	4.017877	10
1840R/C15	C57Bl/6 UUO + ND-13	11	5	8	7	3	5	8	8	14	20	8.9	1.580787	10
13932/DJ1	DJ-1 KO control	5	2	4	1	1	1	3	6	2	3	2.8	0.553775	10
13936/DJ2	DJ-1 KO control	4	1	10	3	4	0	11	4	4	5	4.6	1.097472	10
13937/DJ3	DJ-1 KO control	5	5	3	5	5	6	3	0	4	5	4.1	0.546707	10
13952/DJ4	DJ-1 KO control	0	3	3	4	4	4	0	2	4	15	3.9	1.328742	10
13953/DJ5	DJ-1 KO control	7	6	3	0	3	2	0	6	1	3	3.1	0.795124	10
1339/DJ6	DJ-1 KO UUO + vehicle	24	12	20	30	25	17	30	23	16	11	20.8	2.143725	10
13954R/DJ7	DJ-1 KO UUO + vehicle	28	21	14	62	16	15	15	50	13	20	25.4	5.362835	10
1450R/DJ8	DJ-1 KO UUO + vehicle	26	23	11	13	15	64	45	26	15	18	25.6	5.283517	10
13951R/DJ9	DJ-1 KO UUO + vehicle	34	30	35	27	21	37	22	29	12	36	28.3	2.530042	10
1340R/DJ11	DJ-1 KO UUO + ND13	13	10	26	15	64	52	55	59	48	59	40.1	6.81575	10
13933R/DJ12	DJ-1 KO UUO + ND13	17	23	27	16	13	6	16	6	8	13	14.5	2.187083	10
13934R/DJ13	DJ-1 KO UUO + ND13	63	14	20	28	46	50	55	56	54	36	42.2	5.301572	10
21790R/DJ4	DJ-1 KO UUO + ND13	16	25	14	21	31	17	21	14	23	32	21.4	2.050474	10
21791R/DJ5	DJ-1 KO UUO + ND14	18	23	18	35	26	25	30	10	29	36	25	2.560382	10

Table 2. Individual % area stained positive for F4/80 per microscopy field.

Cortex—% area stained positive for F4/80

Slide Label	Treatment	Field 1	Field 2	Field 3	Field 4	Field 5	Field 6	Field 7	Field 8	Field 9	Field 10	Avg # per Field	SEM	n
1846/C1	C57Bl/6 control	0.30446	0.31171	0.05773	0.08049	0.06757	0.18591	0.09050	0.07980	0.24500	0.33910	0.17623	0.03618	10
1847/C2	C57Bl/6 control	0.15194	0.05809	0.02039	0.02018	0.00180	0.02130	0.02030	0.00240	0.15230	0.05990	0.05086	0.01797	10
1848/C3	C57Bl/6 control	0.19847	0.14318	0.16616	0.16099	0.02664	0.16490	0.12664	0.14847	0.13318	0.12616	0.13948	0.01434	10
1849/C4	C57Bl/6 control	0.18490	0.22664	0.04847	0.14318	0.11616	0.16847	0.18318	0.14616	0.16099	0.02654	0.14047	0.01958	10
1841R/C6	C57Bl/6 UUO + vehicle	1.32497	0.39874	0.59585	1.13051	0.58228	0.58184	0.78228	0.88184	0.69874	0.79585	0.77729	0.08785	10
1842R/C7	C57Bl/6 UUO + vehicle	2.38863	2.33276	2.38274	3.42486	4.37206	2.58763	2.53296	3.72486	3.37306	2.69274	2.98123	0.22128	10
1843R/C8	C57Bl/6 UUO + vehicle	5.54587	5.37382	4.44257	4.82896	5.74729	5.97382	4.14757	4.62896	5.84587	5.34839	5.18831	0.20086	10
1844R/C9	C57Bl/6 UUO + vehicle	0.73163	0.55248	0.86720	0.32478	0.57847	0.22877	0.74463	0.24759	0.72019	0.47788	0.54736	0.07085	10
1836/C11	C57Bl/6 UUO + ND-13	0.66887	0.32327	0.29570	0.28205	0.14368	0.32709	0.25697	0.20542	0.43684	0.49887	0.34388	0.04853	10
1837R/C12	C57Bl/6 UUO + ND-13	0.01422	0.09464	0.10196	0.06743	0.08286	0.09964	0.20196	0.02743	0.01542	0.01486	0.07204	0.01856	10
1838R/C13	C57Bl/6 UUO + ND-13	0.02864	0.04259	0.01942	1.03728	0.09964	0.10196	0.01420	0.07800	0.09940	0.01743	0.15386	0.09883	10
1839R/C14	C57Bl/6 UUO + ND-13	1.67918	1.11608	0.82146	0.53883	2.22886	1.33351	1.35725	1.13794	1.39015	1.24236	1.28456	0.14486	10
1840R/C15	C57Bl/6 UUO + ND-13	1.20605	1.29107	1.91708	0.94834	0.81220	1.64083	1.25003	1.15976	1.06055	1.21073	1.24966	0.10163	10
13932/DJ1	DJ-1 KO control	0.10910	0.25120	0.11010	0.30210	0.20030	0.15840	0.60080	0.48310	0.02540	0.15260	0.23931	0.05668	10
13936/DJ2	DJ-1 KO control	0.16720	0.25710	0.20430	0.29432	0.11320	0.20220	0.11100	0.23410	0.25280	0.25700	0.20932	0.01973	10
13937/DJ3	DJ-1 KO control	0.04420	0.03120	0.06520	0.01130	0.00490	0.02980	0.01990	0.01370	0.01820	0.01526	0.02537	0.00571	10
13952/DJ4	DJ-1 KO control	0.24320	0.20010	0.19990	0.20210	0.22020	0.19899	0.24520	0.34100	0.12110	0.25630	0.22281	0.01776	10
1339/DJ6	DJ-1 KO UUO + vehicle	4.26147	5.84530	2.42755	2.86556	9.97192	6.60343	5.88210	4.26200	5.48350	9.98210	5.75849	0.81887	10
13954R/DJ7	DJ-1 KO UUO + vehicle	10.62744	10.46573	7.45706	11.09432	5.04344	3.35571	11.08900	5.19780	10.64200	7.45010	8.24226	0.92648	10
1450R/DJ8	DJ-1 KO UUO + vehicle	2.86937	7.00368	2.67040	2.00755	2.78249	3.36814	3.35966	2.78240	7.13200	2.87540	3.68511	0.57634	10
13951R/DJ9	DJ-1 KO UUO + vehicle	14.17086	6.67287	9.23641	4.99799	9.69289	10.23100	3.99800	10.30500	14.32400	5.67400	8.93030	1.13677	10
1340R/DJ11	DJ-1 KO UUO + ND13	7.03096	5.11776	5.75066	7.05423	8.27780	7.14679	8.39119	7.27780	6.86786	7.04890	6.99640	0.31259	10
13933R/DJ12	DJ-1 KO UUO + ND13	1.70381	1.98666	4.30951	1.80375	1.89656	5.10951	1.50831	1.86658	4.57310	1.79920	2.65570	0.44424	10
13934R/DJ13	DJ-1 KO UUO + ND13	10.48010	13.87580	15.89226	12.59349	8.86151	10.98479	13.53284	13.98090	9.62895	13.47700	12.33077	0.70863	10
21790R/DJ4	DJ-1 KO UUO + ND13	5.10096	5.69314	10.67483	7.80984	3.18991	3.44576	10.67483	6.80984	3.18991	3.34576	5.99348	0.93038	10
21791R/DJ5	DJ-1 KO UUO + ND14	10.17086	7.67287	9.23641	6.99799	9.94215	14.17086	6.67287	9.23641	4.99799	9.69289	8.87913	0.79333	10
